# Thoracic Endovascular Aortic Repair for Blunt Thoracic Aortic Injury: A Report of Three Cases in Which Surgeries Were Performed at Different Timings

**DOI:** 10.1155/2018/7061509

**Published:** 2018-08-08

**Authors:** Yohei Kawatani, Hirotsugu Kurobe, Yoshitsugu Nakamura, Yuji Suda, Takaki Hori

**Affiliations:** Department of Cardiovascular Surgery, Chiba-Nishi General Hospital, 107-1 Kanegasaku Matsudo-Shi, Chiba-Ken 270-2251, Japan

## Abstract

**Introduction:**

Blunt thoracic aortic injury (BTAI) is a critical condition. Thoracic endovascular aortic repair (TEVAR) is considered a surgical treatment for BTAI. Reports reveal that some patients benefit from conservative and delayed operation rather than emergency operative therapy. Here, we present three BTAI cases that were treated with TEVAR using different timings.

**Case Presentation:**

Case 1 involved a 49-year-old man injured in a car accident and who went into shock. After stabilization with Advanced Trauma Life Support in the emergency room, TEVAR was performed immediately. Case 2 involved a 69-year-old man who was injured after falling. His hemodynamic status was stable and enhanced computed tomography revealed intraluminal hematoma. He underwent TEVAR 15 days after the injury occurred, following conservative therapy. Case 3 involved a 60-year-old man who was injured in a car accident and presented BTAI with subarachnoid hemorrhage and diaphragm tear. A pseudoaneurysm was observed in the distal aortic arch. After open abdominal exploration, diaphragm repair, and observation for subarachnoid hemorrhage, TEVAR was performed 8 hours after arrival. All three patients survived.

**Conclusions:**

We treated BTAI successfully. We suggest that TEVAR is useful for BTAI. The timing of the operation and therapeutic option, including conservative therapy, should be decided for each patient.

## 1. Introduction

Blunt thoracic aortic injury (BTAI) occurs in 2% of blunt trauma patients [1]. BTAI is a critical condition with an overall mortality of up to 80% and a 20% mortality even in treated patients [2].

Five percent of patients with BTAI present with aortic rupture that can cause early death in the acute phase, making early operative management a necessity [3]. However, in many patients with coexisting injury [4], immediate surgery for the aorta is usually infeasible.

Recently, thoracic endovascular aortic repair (TEVAR) has been noted as a better surgical option for emergency conditions involving the aorta. Additionally, it has been demonstrated that some patients benefit from conservative therapy rather than emergency operative therapy based on the type of injury [5].

We report 3 cases treated with TEVAR using different timings because of the type of injury and coexisting organ injury.

## 2. Case Presentation

### 2.1. Case 1

**Figure 1:**
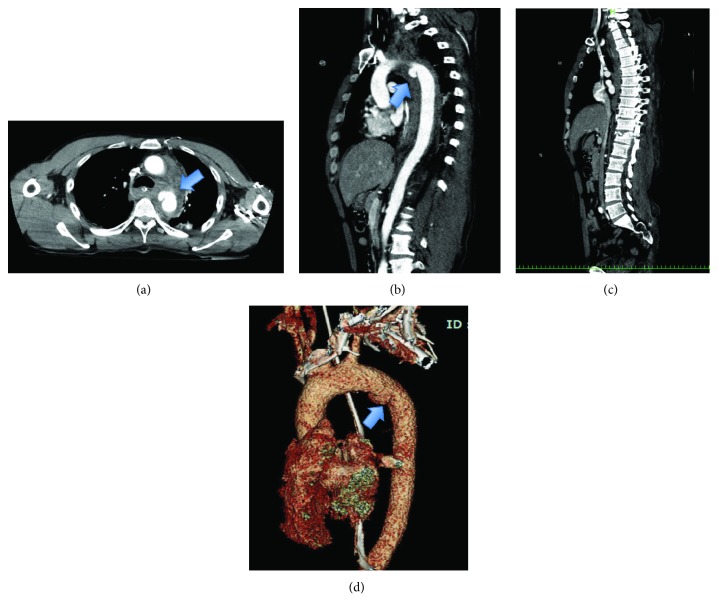
Case 1. Preoperative contrast-enhanced computed tomography: (a) axial, (b) sagittal image, and (d) 3-dimensional image. A pseudoaneurysm was observed in the distal aortic arch (arrow mark). (c) Sagittal image in another slice. Thoracic vertebra 12 and lumbar vertebra 1 are fractured.

A 49-year-old man collided with a car while riding a motorcycle. After the collision, the man was run over by the car. His vital signs were stable on admission, and the patient had no consciousness disorder (blood pressure (BP) 117/56 mmHg; heart rate (HR) 87 bpm; Glasgow coma scale (GCS) E4V5M6). Hematological examination revealed a white blood cell count of 8050/*μ*l, hemoglobin 10.7 g/dl, and platelet 12.4 × 10^9^/l. However, the patient went into shock during care in the emergency room. After volume resuscitation, contrast-enhanced computed tomography (CT) was performed and showed extravasation of the contrast medium and a pseudoaneurysm around the distal arch of the aorta. Additionally, the patient presented with fracture of the Th12 and L1 vertebras, hemothorax, and tear of the right Achilles tendon (Figure 1). A drain was placed in the left thorax, and mechanical ventilation was started under sedation.

**Figure 2:**
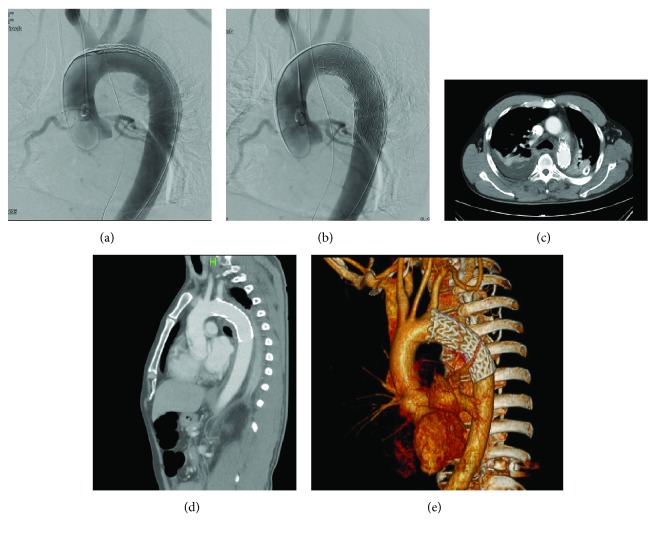
Case 1. Intraoperative aortography: (a) before deploying the stent graft and (b) a pseudoaneurysm was observed but it disappeared after the stent graft was deployed. Postoperative computed tomography: (c) axial image, (d) sagittal image, and (e) 3-dimensional image. The pseudoaneurysm had disappeared.

After these procedures, TEVAR with a 31 × 26 × 100 mm stent graft (Conformable GORE TAG, W. L. Gore & Associates, Newark, DE) was performed successfully. Heparin was not administered during surgery. After the operation, the circulation and respiratory systems were stable. One day after the operation, the patient was weaned from the ventilator without any neurological disorder. Follow-up with enhanced CT showed that the pseudoaneurysm had disappeared (Figure 2). The patient was transferred to a rehabilitation facility without TEVAR-related complications, including any neurological symptoms.

### 2.2. Case 2

**Figure 3:**
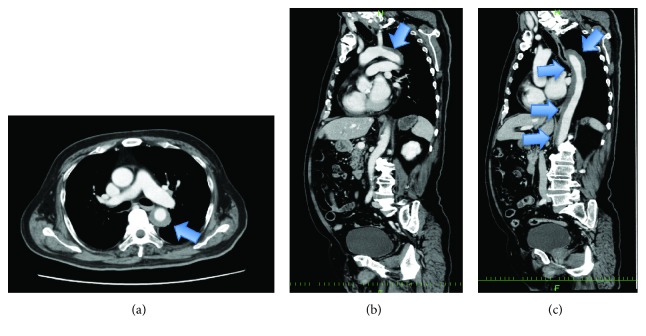
Case 2. Preoperative computed tomography: (a) axial image, (b) sagittal image, and (c) sagittal image in another slice. Intraluminal hematoma was observed (arrows).

A 69-year-old man fell from a ladder. At arrival to the hospital, his vital signs were stable and his consciousness was clear (BP 160/87 mmHg; HR 109 bpm; GCS E4V5M6). He complained of chest and back pain which moved from the shoulder to the chest and back. Enhanced CT was performed which revealed aortic dissection with intramural hematoma. Extravasation and pseudoaneurysm were not observed (Figure 3).

**Figure 4:**
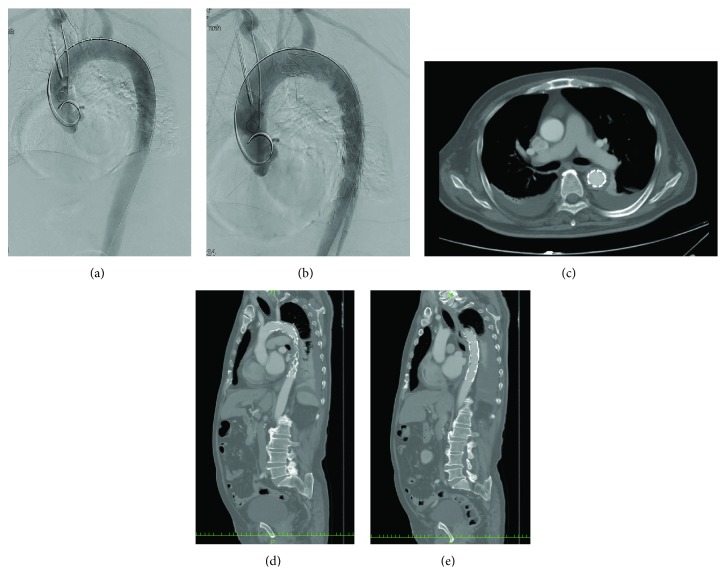
Case 2. Intraoperative aortography: (a) before deploying the stent graft and (b) after deploying the stent graft. There were no endoleaks. Postoperative computed tomography performed one month after the procedure: (c) axial image, (d) sagittal image, and (e) sagittal image in another slice. The thickness of the intraluminal hematoma had decreased.

We commenced conservative therapy which consisted of blood pressure control (target, systolic pressure < 140 mmHg), bed rest for 14 days, and close observation using enhanced and plain CT on hospital days 1, 3, 5, 9, and 14. After this protocol was completed, we performed TEVAR on hospital day 16 as a scheduled operation. We placed 22 × 22 × 100 mm (Valiant Captivia Thoracic Stent Graft, Medtronic, Medtronic, Santa Rosa, CA) and 30 × 26 × 150 mm (Relay Plus, Bolton Medical, Sunrise, FL) stent grafts (Figure 4). During the procedure, we administered heparin with an activated clotting time (ACT) goal of 250 s. At the end of the procedure, heparin was neutralized by an equal amount of protamine.

The patient recovered from anesthesia without any neurological disorder. He was discharged walking, to his home on postoperative day 14, which was hospital day 30. Enhanced CT performed 1 month after the procedure revealed that the thickness of the intraluminal hematoma had decreased (Figure 4).

### 2.3. Case 3

**Figure 5:**
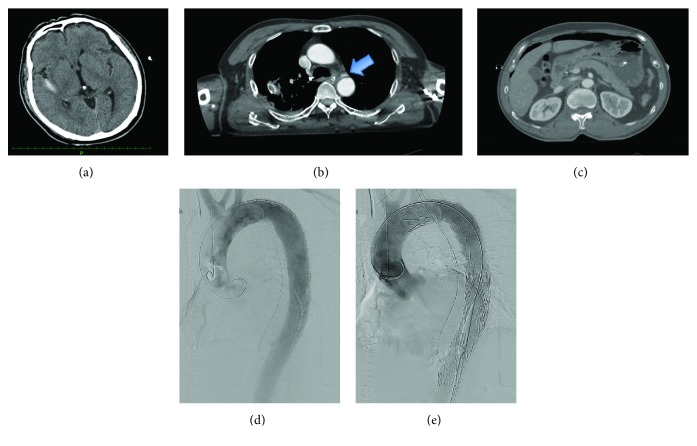
Case 3. Enhanced computed tomography performed in the emergency room after resuscitation: (a) head axial image, subarachnoid hemorrhage was observed; (b) thoracic axial image, pseudoaneurysm was observed in the distal aortic arch (arrow); and (c) abdominal axial image, free air in the abdomen was observed. Intraoperative aortography: (d) before deploying the stent graft and (e) after deploying the stent graft. There were no endoleaks.

A 60-year-old man was hit by a car while walking. His hemodynamic status on arrival to the hospital was stable (BP 120/62 mmHg, HR 85 bpm), but his consciousness was impaired (GCS E1V1M1). After standard Advanced Trauma Life Support with endotracheal intubation, fluid resuscitation, blood transfusion, and CT were performed. Contrast-enhanced CT showed subarachnoid hemorrhage, free air in the abdomen, aortic dissection, and a pseudoaneurysm around the distal arch (Figure 5). However, the patient was hemodynamically stable.

Emergency explorative laparotomy was performed, and a diaphragm tear was observed and repaired. Conservative therapy and close observation were applied for the subarachnoid hemorrhage and BTAI. After 6 hours of observation, CT was performed again and the subarachnoid hemorrhage appeared not to progress. The patient was transferred to the operating room, and TEVAR was performed with a 26 × 22 × 150 mm (Valiant Captivia Thoracic Stent Graft, Medtronic, Medtronic, Santa Rosa, CA) stent graft. During the procedure, heparin was administered with an ACT goal of 250 s and was neutralized after surgery by an equal amount of protamine.

After surgery, the patient's hemodynamics were stable. The patient was returned to the intensive care unit (ICU) on artificial ventilation. He recovered consciousness in the ICU. After extubation, the patient had muscle weakness of both lower limbs which were associated with the TEVAR; however, the weakness disappeared spontaneously. The patient was subsequently discharged to the rehabilitation facility.

## 3. Conclusions

Blunt thoracic aortic injury (BTAI) frequently occurs in the isthmus of the aorta and accounts for 93% of all BTAI [6]. This phenomenon may be explained by the mechanism of injury involving tension between the aortic arch, which is comparably free, and the descending aorta, which is fixed on the thoracic wall [4]. Another possible mechanism is the occlusion of the abdominal aorta caused by increased pressure on the abdomen [7], explaining the coexistence of BTAI with abdominal organ injury [8]. In this report, the lesions in all the presented cases occurred in the isthmus.

Emergency surgery in patients with unstable hemodynamics due to BTAI seems necessary. Even if the hemodynamics are stable, patients who present with extravasation of contrast medium or pseudoaneurysm need prompt transfer to the operation room [9]. Moreover, 81.4% of BTAI patients have coexisting injury [4]. In these patients, the coexisting injury decides the treatment priority and occasionally, surgery for vascular injury cannot be performed immediately. In 50% of the patients who undergo conservative therapy, clinical symptoms were observed and 21% presented with dilatation of the injured aorta [10]. When these occur, surgery is the recommended therapy for the BTAI.

In a report, BTAI was graded I–IV; I: intimal injury, II: intramural hematoma, III: pseudoaneurysm, and IV: rupture. The authors suggested that for grade I, conservative therapy is more effective than surgery. Moreover, for patients with intramural hematoma, delayed surgery was more effective than emergency surgery [5].

In case 1, although the patient's hemodynamics were stable, extravasation was observed. Additionally, BTAI was the injury that was urgently treated. Therefore, we transferred the patient to the operation theater immediately after examination in the emergency room. In case 2, the injury was considered as grade II. We commenced treatment conservatively which is usually applied for patients with acute aortic dissection Stanford type B. After the conservative protocol, we performed the operation.

The timing for delayed surgery is unclear. In acute aortic dissection, regarding the safety of the procedure, complications were more frequently observed in patients who underwent TEVAR within 14 days than in those who underwent surgery between 14 days and 6 months [11]. In addition, Kato et al. reported that TEVAR for type B aortic dissection was effective when the intimal tear, that is, entry for pseudo lumen, was closed within 6 months after onset [12]. We applied this knowledge to our treatment of BTAI and performed delayed operation on day 16. The patient in case 2 had no complications related to BTAI or TEVAR.

In case 3, the patient presented with multiple injuries that needed urgent therapy. The subarachnoid hemorrhage was accorded the highest priority, followed by the free air in the abdomen suggesting injury in the abdominal organs, and finally, the aortic injury. After observation which revealed that the subarachnoid hemorrhage did not worsen, followed by exploratory laparotomy with diaphragm repair, we performed TEVAR. Many guidelines suggest that if the hemodynamics are stable, neurological, respiratory, and abdominal organ injury should be treated first [10].

Regarding the surgical modality for BTAI, endovascular surgery is preferable because it is associated with a lower mortality and morbidity. Overall mortality after surgical repair for BTAI was 23.5% according to the American Association for the Society of Trauma 2 study [13]. However, that of endovascular surgery has been reported as 7.2% [9]. Endovascular surgery has an advantage because it can be performed without aorta clamping, cardiopulmonary bypass, and circulatory arrest. In endovascular surgery, a lesser amount of heparin may be administered and this is more beneficial for patients with coexisting injury. If the patient is fit for TEVAR and the appropriate device is available, endovascular therapy is considered a better operative modality. In the presented cases, we used available devices that were suitable for the patients' anatomical characteristics.

Nevertheless, TEVAR is contraindicated in injury to the aortic arch and ascending aorta. However, most BTAIs occur in the isthmus which is in the distal arch [6]. Therefore, TEVAR may be used for most BTAI. Yet, there are reports where patients with traumatic injury and aneurysm rupture in the aortic arch were treated with TEVAR [14, 15]. Hence, we suggest that TEVAR should be considered even in aortic arch injury, further highlighting the importance of TEVAR in the treatment of BTAI.

In conclusion, we report the successful treatment of BTAI with TEVAR and suggest that TEVAR is a useful treatment option for BTAI. The timing of the surgery and therapeutic options including conservative care and delayed surgery should be decided considering the hemodynamic status, type of aortic injury, and urgency of coexisting injury.

## Abbreviations

BTAI: Blunt thoracic aortic injury
TEVAR: Thoracic endovascular aortic repair
GCS: Glasgow coma scale
ACT: Activated clotting time
ICU: Intensive care unit.
